# Diagnostic accuracy of loop-mediated isothermal amplification (LAMP) for detection of *Leishmania* DNA in buffy coat from visceral leishmaniasis patients

**DOI:** 10.1186/1756-3305-5-280

**Published:** 2012-12-03

**Authors:** Md Gulam Musawwir Khan, Khondaker Rifat Hasan Bhaskar, Md Abdus Salam, Tania Akther, Gerd Pluschke, Dinesh Mondal

**Affiliations:** 1 International Centre for Diarrhoeal Disease Research, Bangladesh (ICDDR,B), Mohakhali, Dhaka, 1212, Bangladesh; 2 Rajshahi Medical College, Rajshahi, 6000, Bangladesh; 3 Head of Department, Medical Parasitology & Infection Biology, Molecular Immunology, Basel, Switzerland

**Keywords:** LAMP, Visceral leishmaniasis, Diagnosis, PCR, Bangladesh

## Abstract

**Background:**

Visceral leishmaniasis (VL) remains as one of the most neglected tropical diseases with over 60% of the world’s total VL cases occurring in the Indian subcontinent. Due to the invasive risky procedure and technical expertise required in the classical parasitological diagnosis, the goal of the VL experts has been to develop noninvasive procedure(s) applicable in the field settings. Several serological and molecular biological approaches have been developed over the last decades, but only a few are applicable in field settings that can be performed with relative ease. Recently, loop-mediated isothermal amplification (LAMP) has emerged as a novel nucleic acid amplification method for diagnosis of VL. In this study, we have evaluated the LAMP assay using buffy coat DNA samples from VL patients in Bangladesh and compared its performance with leishmania nested PCR (Ln-PCR), an established molecular method with very high diagnostic indices.

**Methods:**

Seventy five (75) parasitologically confirmed VL patients by spleen smear microcopy and 101 controls (endemic healthy controls −25, non-endemic healthy control-26, Tuberculosis-25 and other diseases-25) were enrolled in this study. LAMP assay was carried out using a set of four primers targeting *L. donovani* kinetoplast minicircle DNA under isothermal (62 °C) conditions in a heat block. For Ln-PCR, we used primers targeting the parasite’s small-subunit rRNA region.

**Results:**

LAMP assay was found to be positive in 68 of 75 confirmed VL cases, and revealed its diagnostic sensitivity of 90.7% (95.84-81.14, 95% CI), whereas all controls were negative by LAMP assay, indicating a specificity of 100% (100–95.43, 95% CI). The Ln-PCR yielded a sensitivity of 96% (98.96-87.97, 95% CI) and a specificity of 100% (100–95.43, 95% CI).

**Conclusion:**

High diagnostic sensitivity and excellent specificity were observed in this first report of LAMP diagnostic evaluation from Bangladesh. Considering its many fold advantages over conventional PCR and potential to be used as a simple and rapid test in the VL endemic areas of the Indian subcontinent, our findings are encouraging, but further evaluation of LAMP is needed.

## Background

Visceral leishmaniasis (VL) is considered as one of the most neglected tropical diseases. In the Indian subcontinent VL is mainly caused by the intracellular protozoan parasite *Leishmania donovani* and transmitted exclusively by the bite of the sandfly, *Phlebotomous argentipes*. The annual incidence worldwide is approximately 0.2 to 0.4 million cases with mortality rates of 1.5% (93 deaths/6224 VL cases from 2004–2008) in Bangladesh [1]. Surprisingly, over 60% of the world’s VL cases occur in three countries of the Indian subcontinent (India, Bangladesh and Nepal) with 40,000 or more reported cases per year [2].

Diagnosis of VL is still a great challenge and is cumbersome, especially in the resource limited settings of the Indian subcontinent. Definitive diagnosis of VL is of paramount importance to provide a specific treatment regimen as well as in understanding the disease epidemiology. Parasitological confirmation of VL through microscopy still relies on invasive procedure like spleen, bone-marrow or lymph-node aspiration. Involvement of substantial risk and skilled personnel are among the major limitations associated with these procedures, so access to such diagnostic facilities is far beyond the reach of most of the poor people affected with VL living in the remote rural areas.

Efforts are evolving continuously to develop diagnostic methods for VL but existing ones still have some limitations. Serological methods like direct agglutination test (DAT) and rK39 (recombinant k39 antigen-based immunochromatographic strip test) dipstick are now well standardized [3-5], but these tests are only for antibody detection and assessment of cure after treatment and discrimination between symptomatic and asymptomatic cases is not possible. Moreover, cross reactivity with other diseases and inconsistent performance in the case of HIV-VL co-infection are among other major obstacles of the serological tests [6]. Antigen detection from urine through latex agglutination test (KAtex) could be another promising tool for direct diagnosis of VL but poor to moderate sensitivities have been revealed in several studies [7-9] that restrict its use in the field settings. Polymerase Chain Reaction (PCR) has been developed and evaluated as a potent tool for rapid and sensitive detection of *Leishmania* DNA from peripheral blood and buffy coat which is ideal for minimal invasive procedure [10-12]. However, PCR is neither a pragmatic or cost effective method for diagnosis of VL in developing countries such as the Indian subcontinent, as it requires a well-established laboratory and skilled personnel. Recently, a promising diagnostic tool, loop-mediated isothermal amplification (LAMP) has been developed with its potential for not only rapid and sensitive diagnosis but also its feasibility as an alternative technique to conventional PCR method in field conditions [13]. LAMP assay has also been established to detect *Leishmania donovani* DNA from blood samples of VL patients and the results were comparable with that of conventional PCR [14].

This prospective study was designed to evaluate the diagnostic accuracy of LAMP for rapid and sensitive detection of *L. donovani* DNA from buffy coat of confirmed VL patients and to examine its efficacy as a diagnostic alternative to PCR.

## Methods

### Ethical approval

Ethical clearance was obtained from the International Centre for Diarrhoeal Disease Research, Bangladesh (ICDDR, B), ethical review committee. Informed written consent was obtained from each patient or from their legal guardian before splenic aspiration and venipuncture. Written consent was also obtained from all controls before including their samples in the study.

### Patients

A total of 75 confirmed VL patients were enrolled in the study. All of the subjects were admitted to Rajshahi Medical College Hospital (RMCH), Bangladesh from January 2010 to October 2011. The definitive diagnosis of VL was based on the microscopic demonstration of *Leishmania* amastigotes in the splenic aspiration smear.

### Controls

A total of 101 subjects were enrolled in this study as control. The controls were divided into three categories. Twenty five (25) endemic healthy controls were collected from Godagari sub district, a highly endemic area of VL in the Rajshahi division, Bangladesh. Twenty six (26) apparently healthy controls without any signs and symptoms of present VL or past history of VL were also enrolled from VL non endemic areas. Fifty (50) disease controls, including 25 tuberculosis patients confirmed as sputum positive for acid fast bacilli (AFB) by microscopy from the National Institute of Diseases of Chest and Hospital (NIDCH), Mohakhali, Dhaka and another 25 patients with other febrile diseases (Acute Lymphoblastic Leukemia 2, Acute Myeloid Leukemia 1, Aplastic anaemia 1, Chronic Liver Disease 3, Chronic Myeloid Leukemia 3, Enteric fever 2, L. vulguris 1, Liver Abscess 1, Pyrexia of Unknown Origin 3, Rheumatic fever 1, SOL in spleen 1, Thalassemia 5, Viral hepatitis 1) having fever for more than 2 weeks and admitted into different wards of RMCH were also included in the study.

### Case definition

Confirmed VL patients were selected based on splenic smear positive for *L. donovani* amastigote.

### Splenic aspiration and microscopy

Splenic aspiration was performed after excluding contraindications for the procedure in each case by an experienced doctor following standard technique as described by Bryceson *et al.*[15]. In every instance, two good quality smears were prepared by a proficient laboratory technician and stained with Leishman stain. The smear was read in a standardized way under 10 × 100 magnification of a CH-20 Olympus microscope (Model CH20BIMF200, Olympus Optical Co., Ltd. JAPAN) for the presence of *L. donovani* amastigotes by an experienced microscopist at the department of Microbiology of Rajshahi Medical College (RMC). Presence of LD bodies was graded on a scale from 1+ to 6+. If the number of amastigotes counted per field was >100, 10–100 or 1–10, it was graded as 6+, 5+ and 4+ respectively. Similarly, 1–10 amastigotes in 10, 100 or 1000 fields were graded as 3+, 2+ and 1+ respectively [15]

### Buffy coat preparation

Maintaining good clinical practice, 3.0 mL of venous blood was collected in vacuette (K3 EDTA tube) from all subjects. Immediately after collection, blood was centrifuged by density gradient centrifugation with an equal volume of Histopaque 1119 (Sigma Aldrich, USA). After centrifugation, plasma and buffy coat samples were transferred into two different sterile 1.5 mL micro-centrifuge tubes and stored at −20°C at RMC until shipment to ICDDR, B. The samples were transferred periodically to ICDDR, B maintaining cold chain.

### rK39 RDT

For all the subjects, rK39 was performed from plasma samples according to manufacturer’s instruction provided as product inserts (the Kalazar Detect, InBios, Seattle, WA, USA).

### DNA extraction

DNA was extracted from buffy coats using the commercially available QIAamp DNA Blood Mini Kit (Qiagen, Hilden, Germany) according to the manufacturer’s instructions. After extraction, DNA was eluted in a final volume of 0.2 mL of AE buffer (Qiagen Hilden, Germany). Purity of DNA was measured in a Nano-drop (~5μL of DNA) and was found to be satisfactory as an OD ratio at A260/A280 was within the range of 1.7-1.9 in all DNA samples.

### Leishmania nested PCR (Ln-PCR)

For detection of parasite DNA, *Leishmania* specific nested PCR (Ln-PCR) was performed with primers targeting the parasites' small-subunit rRNA (*SSU*-rRNA) region with some minor modifications as described in a previous study [11]. For both the primary and nested cycles, 2 μL of DNA was amplified in a final volume of 25 μL using the commercially available kit from GE Healthcare (UK) which contained 200 μM each dNTP in 10 mM Tris–HCl, 50 mM KCl and 1.5 mM MgCl_2,_ 2.5units of pure Taq DNA polymerase, BSA and stabilizers for each reaction. Prior to the second amplification or nested PCR, the amplified products from the first run were diluted at 1:20 with molecular grade water. An additional 2.0 mM MgCl_2_ was added to the final reaction mixture prior to the second round of amplification. The final amplicons were visualized as 358 bp on 1.5% agarose gel.

For the second round of amplification, 35 cycles were used consisting of denaturation at 94°C for 30 s, 65°C for 30 s, and 72°C for 30 s followed by a final extension at 72°C for 5 min, in a BioRad’s MyCycler.

### Loop-mediated isothermal amplification (LAMP)

**Figure 1:**
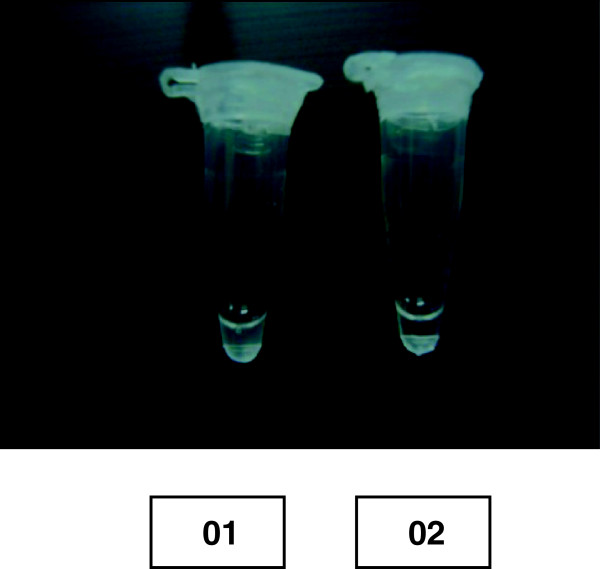
Visualization of LAMP products, 01 = A patient sample found positive as indicated by turbidity, 02 = A control found negative as indicated by absence of turbidity.

The LAMP reaction was carried out according to the original reports described by Tagaki *et al.*[14] with minor modifications. Initially a primer mix was prepared containing 40 pmol of each of FIP and BIP, and 5 pmol of each of F3 and B3c primers. The final reaction mixture of 25 μL contained 0.9 μL of primer mix, 1.4 mM of each deoxynucleoside triphosphate, 0.8 M betaine and 2.5 μL of 10X ThermoPol Reaction Buffer (New England Biolabs, Ipswich, MA), containing 20 mM Tris–HCl, 10 mM (NH_4_)_2_SO_4_, 10 mM KCl, 2 mMMgSO_4_, 0.1% Triton X-100, pH 8.8, 25°C at final 1X and an additional 6 mM MgSO_4_ and 8 units of *Bst* polymerase large Fragment (New England Biolabs, Ipswich, MA), and 2 μL of buffy coat DNA. Each tube containing the reaction mixture was sealed with paraffin (as LAMP causes massive amplification which might result in contamination of DNA) and incubated in a heat block at 62°C for 1 hour and 30 min. During the course of the reaction, the binding of a reaction by-product (pyrophosphate ions) to magnesium ion results in a white precipitate, entailing the reaction fluid turbid. At the end of incubation, turbidity was measured visually and the results were interpreted as positive or negative based on turbidity (Figure 1).

### Analytical sensitivity of LAMP

**Figure 2:**
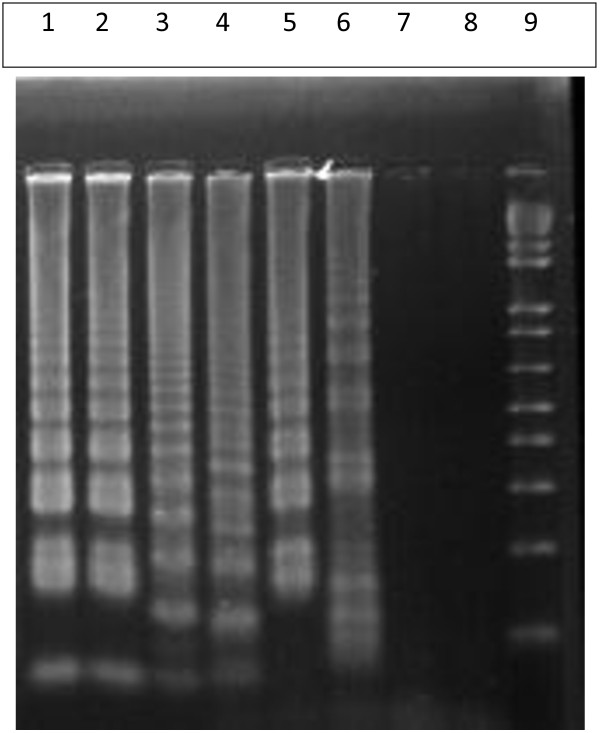
**Sensitivity of LAMP reaction against serial dilution of cultured promastigote DNA seen in 1.5% agarose gel electrophoresis.** Lanes 1 to 7 contain cultured promastigote *L. donavani* DNA of 100 pg, 10 pg, 1 pg, 100 fg, 10 fg, 1 fg and 0.1 fg respectively; Lane 08 = negative control (PCR grade water) and Lane 09 = 100 bp ladder.

**Figure 3:**
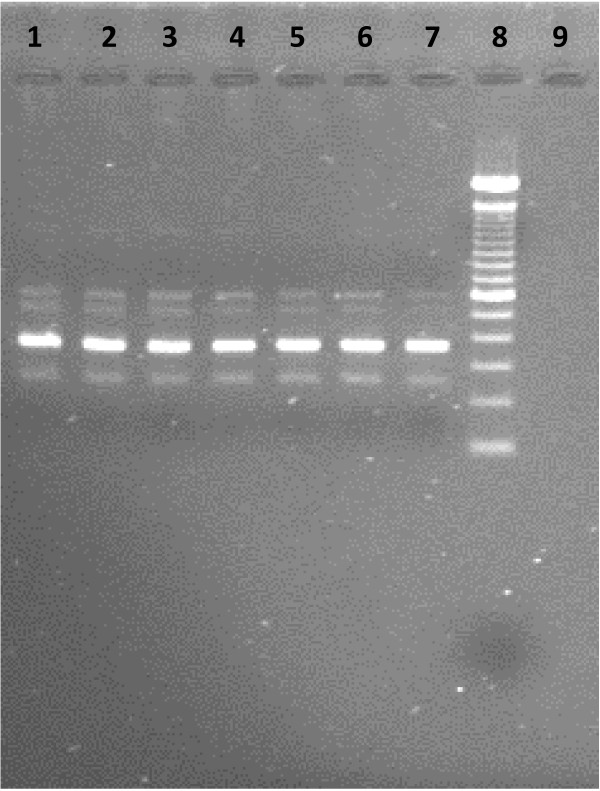
**Sensitivity of Ln-PCR against serial dilution of cultured promastigote DNA seen in 1.5%agarose gel electrophoresis.** Lanes 1 to 7 contain cultured promastigote *L. donovani* DNA of 100 pg, 10 pg, 1 pg, 100 fg, 10 fg, 1 fg and 0.1 fg respectively; Lane 08 = 100 bp ladder and Lane 09 = negative control (PCR grade water)

In order to compare the sensitivity of the LAMP assay, *in vitro* promastigote culture (*L. donovani* MHOM/IN/80/DD8) maintained in *Novy-MacNeal-Nicolle medium* (NNN medium) by subpassaging was utilized for extraction of DNA. After extraction, the concentration of DNA was measured by Nano-drop. A serial dilution of cultured promastigote DNA starting from 100 pg to 0.1 fg were prepared in TE buffer (Qiagen Hilden, Germany). The dilutions were amplified by both LAMP and Ln-PCR and products were visualized by agarose gel electrophoresis (Figure 2 & Figure 3).

### Statistical analysis

Sensitivity and specificity were calculated using the online Clinical Calculator 1. Data were entered and clinical parameters were analyzed using the SPSS software (version 11.5) for Windows. Sensitivities of LAMP and Ln-PCR were compared by McNemar’s test using a online calculator (http://www.vassarstats.net/propcorr.html).

## Results

Clinical parameters of the VL patients and controls are shown in Table 1. Among 75 parasitologically confirmed VL cases, 26 were female (34.67%) and 49 were male (65.33%). The mean age of the VL patients was 17.8 years (14.29-21.31, 95% CI) and that of the controls was 26.88 years (24.24-29.52, 95% CI).

**Table 1:** Clinical parameters of cases and controls

**VL cases**		
	**Number**	**Percentage%**
Male	49	65.33
Female	26	34.67
Age	1 y- 60 y (mean 17.80 years)	NA
**Controls**		
Male	70	69.31
Female	31	30.69
Age	2 y- 70 y (mean 26.88 years)	NA
**Clinical signs and symptoms of VL cases**		
Duration of fever (in weeks)	4-52 weeks (mean = 20 weeks)	
Anaemia	74	98.7
Weight Loss	75	100
Hepatomegaly	58	77.3
Splenomegaly	74	98.7
Blackening	72	96.0
Jaundice	1	1.3
Bleeding	6	8.0
Pancytopenia	29	38.7
ESR	25-175 (mean = 102.81)	NA

### Laboratory findings

#### Splenic smear microscopy

All the cases were confirmed to be true VL by the demonstration of *Leishmania* amastigotes in the spleen smear by microscopy (Table 2).

**Table 2:** Sensitivity and Specificity of different methods for diagnosis of VL

**Diagnostic Method**	**Sensitivity in VL group (n = 75),%(95%CI)**	**Specificity in control groups, (n = 101),%(n)**				
		**Endemic Healthy Control (n = 25),%(95%CI)**	**Non-endemic Healthy Control (n = 26),%(95%CI)**	**Other Disease Control (n = 25),%(95%CI)**	**Tuberculosis control (n = 25),%(95%CI)**	**Specificity Total group, (n = 101),%(95%CI)**
**rK39 ICT**	**98.67**	**100**	**100**	**100**	**100**	**100**
	**(99.93-91.78)**	**(100–95.43)**	**(100–95.43)**	**(100–95.43)**	**(100–95.43)**	**(100–95.43)**
**Ln-PCR**	**96**	**100**	**100**	**100**	**100**	**100**
	**(98.96-87.97)**	**(100–95.43)**	**(100–95.43)**	**(100–95.43)**	**(100–95.43)**	**(100–95.43)**
**LAMP**	**90.7**	**100**	**100**	**100**	**100**	**100**
	**(95.84-81.14)**	**(100–95.43)**	**(100–95.43)**	**(100–95.43)**	**(100–95.43)**	**(100–95.43)**

#### rK39 rapid test

All but one patient was found to be positive by rK39 RDT, indicating its sensitivity as 98.7% (99.93-91.78, 95% CI), while all controls were found negative, yielding a specificity of 100% (100–95.43, 95% CI). (Table 2).

#### Ln-PCR and LAMP

Among 75 confirmed VL cases, 72 were found to be positive by Ln-PCR and all the controls were negative, resulting in a sensitivity of 96% (98.96-87.97, 95% CI) and specificity of 100% (100–95.43, 95% CI). Whereas, 68 of 75 VL cases were found to be positive by LAMP and all the controls gave negative results, which yielded a sensitivity of 90.7% (95.84-81.14, 95% CI) and a specificity of 100% (100–95.43, 95% CI) (Table 2). All the laboratory findings are summarized in Table 2.

Further, LAMP assay was able to detect 66 of 72 Ln-PCR positive cases and an additional 2 cases were found positive by LAMP were negative by Ln-PCR.

#### Analytical sensitivity of LAMP

All the serially diluted samples were assessed for the presence of promastigote DNA by both LAMP and Ln-PCR. Ln-PCR was able to detect up to 0.1 fg of DNA against up to 1 fg detected by LAMP assay (Additional file 1).

#### Comparison of different methods

We compared the sensitivity and specificity between Ln-PCR and LAMP assessed by the McNemar test. Comparison of PCR results of VL samples between LAMP and Ln-PCR indicated no significant difference (P value =0.28). As all the controls were negative by both the methods, the McNemar test was not performed for specificity.

## Discussion

Loop-mediated isothermal amplification originally developed by Notomi *et al.*[13] is a less expensive assay which has now been utilized for the development of a number of diagnostic methods to detect several African trypanosome species [16-18], as well as malaria, tuberculosis, and filaria [19-23]. Despite VL being one of the most important parasitic diseases, it still lacks a simple, low-cost and sensitive diagnostic method. Therefore the VL endemic regions in South Asia including Bangladesh requires a definitive diagnostic method that can be performed as a rapid test for VL patients living in immense poverty.

In this study, we have validated the LAMP assay developed by Takagi *et al.*[14], with 75 confirmed VL cases and 101 controls. It has revealed very encouraging results compared to nested PCR assay which is being used widely for the diagnosis of leishmaniasis. In a study conducted by Takagi *et al.*[14], diagnostic sensitivity of LAMP was evaluated for 10 confirmed VL patients and 08 (80%) were found to be positive for parasite DNA. In another study conducted with 30 confirmed VL patients, a sensitivity of 83% and specificity of 98% were reported [24]. To compare the sensitivity and specificity of LAMP using buffy coat DNA in our study consisting of 75 confirmed VL cases and 101 controls, we found a higher sensitivity of 90.7% and specificity of 100%. A similar range of sensitivity was reported by a number of investigators using either whole blood or buffy coat (PBMC) for PCR assays targeting the ITS or mini-exon regions for DNA detection [25-27].

Although in this study we observed the performance of LAMP to be slightly lower than Ln-PCR (sensitivity 96% and parasite detection rate is 0.1 parasite against 1 parasite by LAMP), yet it achieved better results than shown in two previous studies. The reason for this could be the use of buffy coat instead of whole blood. The specificity of the LAMP assay has been shown to be very high as the assay utilizes four sets of primers targeting six distinct target DNA sequences [16,28,29]. In our study, although LAMP has failed marginally to satisfy the WHO sensitivity level of >95% for any acceptable test there are still several advantageous aspects of the LAMP assay that need to be considered.

The very exciting features of LAMP assay include requirement of just a heat block or even a simple water bath instead of a costly thermal cycler, UV gel documentation and transilluminator, which all are essential components of conventional PCR [20,24,30,31]. Moreover, the assay is performed under isothermal conditions at a temperature range of 60°-65°C, circumventing the time length involved in thermal changes of conventional PCR and also prevents or lowers inhibition observed in its later stages [28,32]. However, when the LAMP reaction is performed in a water bath the chances of contamination tends to increase more and hence it is recommended to wrap the cap of the tube with paraffin prior to performing the LAMP assay. Further, the requirement of electrical power supply to operate the water bath at the resource limited settings can be avoided by the usage of alternative power sources such as battery, exothermal chemicals and solar power [31,32].

In addition, results of LAMP assay are interpreted by observing turbidity [20,31,32] that reduces cost and time of post conventional PCR analysis [16,24,28] as well as eliminating the chance of contamination involved in agarose gel electrophoresis [28]. It is also noted that gel electrophoresis involves handling of potent carcinogenic agents such as ethidium bromide which poses a potential threat for those handling it in the laboratory.

Furthermore, the *Bst* polymerase enzyme used in LAMP assay is active at relatively high temperatures, which minimizes the possibility of non-specific priming [16] and is more resistant to inhibitors that prevent conventional PCR [19]. Alongside, the isothermal condition of the assay lowers inhibition observed in the later stages of conventional PCR [29].

Several reports have indicated the usefulness of using heat-treated samples as a template DNA source without compromising the sensitivity, and thus eliminating the need for DNA extraction which reduces both time and cost [16,19,28,31]. Reports from a previous study indicated that LAMP assay for malaria diagnosis using heat treated blood was priced between US$0.4 and US$0.7, that is lower than currently available RDT's [19]. However, in this study we were not able to validate our assay with heat-treated blood samples for the diagnosis of VL. We speculate that, validation of heat treated buffy coat as a DNA source could further minimize the cost for diagnosis if it can be established for leishmaniasis. Further, there is scope to improve the sensitivity of LAMP in the diagnosis of VL if some unexpected problems associated with sample storage and transportation can be minimized. In our case, we collected VL samples from RMCH and stored them there for a certain length of period, this site is about 300 km away from ICDDR, B. Had these steps been avoided, we believe that the sensitivity of LAMP in our study might be slightly increased.

## Conclusion

To achieve the goal of a VL elimination program, development and introduction of rapid and less expensive diagnosis through non-invasive or minimally invasive clinical samples has no alternative and LAMP method has the potential to full-fill this requirement. So we recommend LAMP as a future novel VL diagnosis tool in the field condition, but before being introduced in the field further evaluation is necessary with larger case and control groups using heat-treated blood samples.

## Abbreviations

VL: Visceral leishmaniasis; LAMP: loop-mediated isothermal amplification; Ln-PCR: Leishmania nested PCR.

## Competing interests

We declare that we have no competing interests. The funding body had no role in study design, data collection and analysis, preparation of the manuscript or decision to publish.

## Authors' contributions

Study was designed by MGMK and DM. Sample collection spleen smear microscopy and clinical examinations were done by MAS. Laboratory experiments were done by KRHB, MGMK and TA. All authors equally contributed in drafting and revising the manuscript. All authors read and approved the final version of the manuscript.

## Supplementary Material

Additional file 1 STARD checklist for reporting of studies of diagnostic accuracy. Click here for file
